# Supplementary material for: Synaptotagmin-1-dependent phasic axonal dopamine release is dispensable for basic motor behaviors in mice
Source: Nat Commun. 2023 Jul 11;14:4120. doi: 10.1038/s41467-023-39805-7 (PMC10336101; doi:10.1038/s41467-023-39805-7)
Supplement: Supplementary file 3 — Reporting Summary [file 41467_2023_39805_MOESM3_ESM.pdf]

nature portfolio

Corresponding author(s): Trudeau, Louis-Eric

Last updated by author(s): May 5 2023

Reporting Summary

Nature Portfolio wishes to improve the reproducibility of the work that we publish. This form provides structure for consistency and transparency in reporting. For further information on Nature Portfolio policies, see our Editorial Policies and the Editorial Policy Checklist.

Statistics

For all statistical analyses, confirm that the following items are present in the figure legend, table legend, main text, or Methods section.

n/a	Confirmed	
☐	☒	The exact sample size (*n*) for each experimental group/condition, given as a discrete number and unit of measurement
☒	☐	A statement on whether measurements were taken from distinct samples or whether the same sample was measured repeatedly
☐	☒	The statistical test(s) used AND whether they are one- or two-sided<br>*Only common tests should be described solely by name; describe more complex techniques in the Methods section.*
☒	☐	A description of all covariates tested
☐	☒	A description of any assumptions or corrections, such as tests of normality and adjustment for multiple comparisons
☐	☒	A full description of the statistical parameters including central tendency (e.g. means) or other basic estimates (e.g. regression coefficient) AND variation (e.g. standard deviation) or associated estimates of uncertainty (e.g. confidence intervals)
☐	☒	For null hypothesis testing, the test statistic (e.g. *F*, *t*, *r*) with confidence intervals, effect sizes, degrees of freedom and *P* value noted<br>*Give P values as exact values whenever suitable.*
☒	☐	For Bayesian analysis, information on the choice of priors and Markov chain Monte Carlo settings
☒	☐	For hierarchical and complex designs, identification of the appropriate level for tests and full reporting of outcomes
☒	☐	Estimates of effect sizes (e.g. Cohen's *d*, Pearson's *r*), indicating how they were calculated

Our web collection on statistics for biologists contains articles on many of the points above.

Software and code

Policy information about availability of computer code

Data collection	Clampex and Clampfit, version 10.7 (Axon Instruments .abf files) for voltammetry. Superflex sensor version 4.6 (Omnitech) using the Fusion software (v.5.6 Superflex edition) for open field behavior analysis. Image Quant LAS 4000 software for western blot. Expression suite software (Thermo Fischer Scientific) for qRT-PCR. ESA CoulArray v.3.0 and Chromaleon v.7.2.1 software for HPLC. Stereo Investigator (MBF BioScience) for stereology.
Data analysis	Image J, version 1.53 (National Institutes of Health) for all acquired images. GraphPad Prism version 9 for generation graphs and statistical analyses.

For manuscripts utilizing custom algorithms or software that are central to the research but not yet described in published literature, software must be made available to editors and reviewers. We strongly encourage code deposition in a community repository (e.g. GitHub). See the Nature Portfolio guidelines for submitting code & software for further information.

Data

Policy information about availability of data

All manuscripts must include a data availability statement. This statement should provide the following information, where applicable:

- Accession codes, unique identifiers, or web links for publicly available datasets
- A description of any restrictions on data availability
- For clinical datasets or third party data, please ensure that the statement adheres to our policy

Source data are provided with this paper in the Source Data File document. Access to original raw images and electrochemistry recording traces is available on

request from the corresponding author.

Human research participants

Policy information about studies involving human research participants and Sex and Gender in Research.

Reporting on sex and gender	N/A
Population characteristics	N/A
Recruitment	N/A
Ethics oversight	N/A

Note that full information on the approval of the study protocol must also be provided in the manuscript.

Field-specific reporting

Please select the one below that is the best fit for your research. If you are not sure, read the appropriate sections before making your selection.

[x] Life sciences [ ] Behavioural & social sciences [ ] Ecological, evolutionary & environmental sciences

For a reference copy of the document with all sections, see nature.com/documents/nr-reporting-summary-flat.pdf

Life sciences study design

All studies must disclose on these points even when the disclosure is negative.

Sample size	Sample sizes were comparable or higher to those typical in the field and were determined to provide sufficient power to detect effect sizes of at least 20%.
Data exclusions	Data were only excluded in the case of experimental failure (ex: if a construct failed to express, that entire preparation was not used) and never purely to remove outliers or otherwise "clean" data.
Replication	Sample sizes in the present study were between 4 and 18 and are specified in the figure legends. All attempts at replication were successfull. Some experimental results of the present manuscript replicate findings from Banerjee et al. 2020 (DOI: 10.7554/eLIFE.58359) including voltammetry and microdialysis data. Some divergent results are described in the manuscript.
Randomization	Animals were alocated to experimental groups based on their genotype (WT, HT or KO), but once experimental groups were established, the data were acquired blinded to the genotype.
Blinding	All acquired data were blinded regarding the genotype. The investigators were also blinded to group allocation during data analysis. For behavioral experiments, saline or treatment were given unblinded but to unknown genotypes.

Reporting for specific materials, systems and methods

We require information from authors about some types of materials, experimental systems and methods used in many studies. Here, indicate whether each material, system or method listed is relevant to your study. If you are not sure if a list item applies to your research, read the appropriate section before selecting a response.

Materials & experimental systems

n/a	Involved in the study	
[ ]	[x]	Antibodies
[x]	[ ]	Eukaryotic cell lines
[x]	[ ]	Palaeontology and archaeology
[ ]	[x]	Animals and other organisms
[x]	[ ]	Clinical data
[x]	[ ]	Dual use research of concern

Methods

n/a	Involved in the study	
[x]	[ ]	ChIP-seq
[x]	[ ]	Flow cytometry
[x]	[ ]	MRI-based neuroimaging

Antibodies

Antibodies used	mouse anti-TH, 1:2000, MAB318, Millipore Sigma, USA.

rabbit anti-TH, 1:1000, AB152, Millipore Sigma, USA.
rabbit anti-Syt1, 1:1000, 105-103, Synaptic Systems, Germany.
rabbit anti-VMAT2, 1:2000, gift from Dr. Gary Miller, Columbia University, USA.
mouse anti-MAP2, 1:2000, MAB3418, Millipore Sigma, USA.
rat anti-DAT, 1:1000, MAB 339, Clone DAT-Nt, Millipore Sigma, USA.
mouse anti-beta-actin, 1:5000, A3854, Sigma-Aldrich, Canada
chicken anti-GFP, 1:1000, GFP-1020, Aves Lab, USA.
rabbit anti-RFP, 1:1000, CA600-401-379, Rockland Inc, USA.
rabbit anti-5HT, 1:2000, 20080, Immunostar, IN, USA

Validation

The two primary anti-TH antibodies are broadly used and their specificity is validated by the presence of a strong signal that is selectively detected in regions such as the striatum. They also label a single band in western blot experiments, as shown in our figure S7. The specificity of the Syt1 antibody is demonstrated by the absence of signal in KO tissue (https://pubmed.ncbi.nlm.nih.gov/31501440/). The specificyt of the VMAT2 antibody has been demonstrated previously (https://www.pnas.org/doi/full/10.1073/pnas.1402134111). The rat anti-DAT antibody is broadly used and its specificity is validated by the presence of strong signal only in regions of the brain containing dopamine neurons or their axon terminals (https://www.emdmillipore.com/CA/en/product/Anti-Dopamine-Transporter-Antibody-NT-clone-DAT-Nt,MM_NF-MAB369?ReferrerURL=https%3A%2F%2Fwww.google.ca%2F). The mouse beta actin antibody is broadly used and labels a single band in western blots, demonstrating its specificity (https://www.sigmaaldrich.com/CA/en/product/sigma/a3854). The chicken anti-GFP antibody is broadly used (https://www.aveslabs.com/products/anti-green-fluorescent-protein-antibody-gfp) and its specificity in our experiments is valided by the fact that it selectively detected the ChR2-eYFP construct that we expressed in dopamine neurons after viral labelling of dopamine neurons (figure 2). The anti-RFP antibody's specifity is demonstrated by its selective detection of neurons infected with the TH-CRE-fusionred virus (figure 9). It has also been used in a large number of previous publications (https://www.rockland.com/categories/primary-antibodies/rfp-antibody-pre-adsorbed-600-401-379/). The anti-5HT antibody has been previously validated to selectively label 5-HT containing neurons of the raphe nuclei (https://www.immunostar.com/product/5-ht-serotonin-rabbit-antibody/).

Animals and other research organisms

Policy information about studies involving animals; ARRIVE guidelines recommended for reporting animal research, and Sex and Gender in Research

Laboratory animals

The Syt1-floxed mouse line (Syt1lox/lox) was obtained from Dr. Schneggenburger who rederivated the Syt1tm1a(EUCOMM)Wtsi (EMMA, Monterotondo, Italy; EM06829; RRID:MGI_5450372) strain with a C57Bl6 background carrying a constitutively expressing FLP gene in order to remove the Frt-flanked lacZ/neo insert84. We then crossed the Syt1lox/lox mice with B6.SJL-Slc6a3tm1.1(cre)Bkmm/J; DATIRES-Cre mice (The Jackson Laboratory, stock 006660, USA), driving expression of the cre recombinase under the control of the DAT promoter, resulting in the selective deletion of Syt1 alleles in DA neurons (Fig. 1A). Syt1-floxed/DATIRES-cre mice were bred from heterozygous crosses to generate Syt1 conditional knockout in DA neurons (cKODA), heterozygotes and wild-type animals, referred in the manuscript as Syt1-/-, Syt1+/- and Syt1+/+ respectively. Animals were used at ages between 6 and 12 weeks, as specified in the manuscript. For cultures, pups were used between the date of birth and day 3 after birth.

Wild animals

No wild animals were used in the study.

Reporting on sex

Male and female were used in this study and their proportion (M/F) is reported for each experiment. However, the number of mice from each sex did not allow to separately analyze male and female in statistical analyses.

Field-collected samples

The study did not involve field-collected samples.

Ethics oversight

The experimental protocols were approved by the animal ethics commitees (CDEA) of the Université de Montréal. For microdialysis experiments, the study was approved by the animal care committee of the research center of the Hôpital du Sacré-Cœur-de-Montréal.

Note that full information on the approval of the study protocol must also be provided in the manuscript.
